# Influence of Multiple Recycling Cycles on the Mechanical, Rheological and Thermal Behaviour of a Commercial Cellulose Acetate Blend

**DOI:** 10.3390/polym18070858

**Published:** 2026-03-31

**Authors:** Iman Taha, Lara Trussina-Miltz

**Affiliations:** 1 Institute for Sustainable Polymers and Composites (iSPC), Aalen University of Applied Sciences, 73430 Aalen, Germany; 2 Department of Design and Production Engineering, Faculty of Engineering, Ain Shams University, Cairo 11517, Egypt

**Keywords:** biopolymer, cellulose acetate, mechanical recycling, mechanical testing, rheological testing

## Abstract

In this study, the commercial biobased cellulose–acetate-based blend ARBOBLEND^®^ 4655V was analysed with regard to its property changes after multiple mechanical recycling at three different processing temperatures (170 °C, 190 °C, and 210 °C). The results demonstrate that the material properties evolve in a distinct manner across the recycling cycles, depending on the processing temperature. While no relevant changes in the zero-shear viscosity, rheologically indicated average molecular weight, or thermal and mechanical properties were observed at 170 °C, moderate changes were observed at 190 °C, in particular an increase in the zero-shear viscosity and rheological indications of an increased average molecular weight, a broadening of the molecular weight distribution, and a change in crystallisation behaviour over the recycling passes. At 210 °C, a marked reduction in the zero-shear, rheological indications of a decreased average molecular weight, and a narrowing of the molecular weight distribution were observed. These rheology-based structural changes had an effect on the mechanical properties, such as the impact strength, the elongation at break, and the elongation at ultimate tensile stress, which were found to decrease with an increasing number of recycling passes. The study suggests that the processing temperature exerts a significant influence on the recycling behaviour of the cellulose–acetate-based blend ARBOBLEND^®^ 4655V. It is evident that even minor fluctuations in temperature can result in substantial changes to the physical, thermal, and mechanical properties of biopolymers. The findings underline the necessity of conducting recycling-related investigations with the processing temperature as a critical factor, particularly for temperature-sensitive materials such as cellulose–acetate-based compounds.

## 1. Introduction

The market size for polymers was 712 billion U.S. dollars in 2023, thus indicating their substantial economic importance [1]. Polymers are distinguished by their extensive range of modification options, which renders them adaptable to the specific requirements of an application to an unprecedented degree. This adaptability is a key advantage over other materials [2]. Consequently, plastics have a ubiquitous presence across diverse industrial sectors, encompassing vehicles, household goods, medicine, construction, and, most notably, the packaging industry [3]. However, these positive effects are overshadowed by negative aspects, including climate damage and resource intensity.

The longevity of plastics poses a significant challenge to waste management [4]. For instance, since 1995, the amount of packaging waste in Germany has increased from 1.5 million tonnes (~18 kg per capita) to 3 million tonnes in 2023 (~41 kg per capita) [5]. In this context, biopolymers are regarded as a promising alternative to fossil-based plastics, due to their utilisation of renewable raw materials, their potential biodegradability, and their versatility [5]. Consequently, there has been a steady increase in demand for biobased and biodegradable polymers [6]. According to a recent market analysis conducted by the European Bioplastics in cooperation with the Nova Institute [7], the global production volume of biopolymers is approximately 0.5% of the nearly 400 million tonnes of plastic produced annually. The forecast for the subsequent years indicates an increase from approximately 2.47 million tonnes in 2024 to around 5.7 million tonnes in 2029. The average annual growth rate of biopolymers is approximately 17%, which is considerably higher than the 2–3% overall growth of polymers [8].

The term ‘biopolymers’ refers to a class of polymers that are either biobased, biodegradable, or feature both properties, as defined by the European Bioplastics Association [9]. With regard to biobased but not biodegradable materials, polyamide with 14.4% is the most produced biopolymer, closely followed by polytrimethylene terephthalate (13.2%) and polyethylene (11.0%). Regarding biobased and biodegradable plastics, polylactic acid (PLA) and polyhydroxyalkanoates (PHA) have the largest market shares with 42.3% and 17.0%, respectively. Further plastics of this group are cellulose derivatives (3.3%), starch-containing polymer compounds (2.4%), and polybutylene adipate-co-terephthalate (2.0%) [7].

Despite the biodegradability of certain biopolymers, they do not offer a comprehensive solution to the problem of waste management. It is notable that certain biopolymers require a long period of time to undergo degradation. Even cellulose acetate (CA), which is considered relatively rapidly biodegradable, has been shown to degrade at rates ranging from 47 days under laboratory conditions to 81–495 days in a tropical forest atmosphere [10]. However, the degradation rates of these polymers are strongly dependent on polymer structure, degradation methods employed, and environmental influences. This renders the prediction of the exact time of complete degradation in nature a challenging task [11]. A study by Yadav et al. [12] demonstrates the significant impact of the structure–environment-degradation relationships, using CA as a case study. In this case, the biodegradation rate was observed to be highly influenced by the degree of acetyl substitution (DS). In addition, the molecular weight, crystallinity, physical form, impurities, and electron acceptors have been shown to influence the degradation rate. It is evident that environmental factors such as temperature, humidity, pH value, sunlight, and the availability of oxygen, nutrients, and microorganisms also exert an influence. It is imperative to establish an environment that can facilitate partial deacetylation of CA with a high DS, as this is a prerequisite for the subsequent degradation process. However, it should be noted that nature does not provide such ideal conditions.

Consequently, the potential for reducing waste through recycling should also be explored in the context of biodegradable polymers [5]. Plastics recycling can be approached in one of three main ways: thermal recycling (also termed incineration), chemical recycling, and mechanical recycling (subdivided into primary and secondary recycling) [13]. A recent study on the material flow pattern of plastics in Germany in 2023 [14] revealed that a total of 5.91 million tonnes of plastic waste was generated that year, of which approximately 61.1% was incinerated for energy recovery, while 37.9% was mechanically recycled. In Europe, it is estimated that only 18.5% of European plastic production is mechanically recycled, while the remainder is incinerated or landfilled [15]. Globally, the numbers are even more worrying. In 2019, 46% of the plastic waste was sent to landfills, 17% was incinerated, 22% was mismanaged and littered, and only 15% was collected for recycling (OECD Global Plastic Outlook 2022) [16]. Accordingly, the OECD estimates that only 9% of plastics were successfully recycled, taking recycling losses into consideration [17].

Mechanical recycling is a widely utilised recycling method. It is evident that the mechanical recycling process has the potential to induce thermal and mechanical damage to the polymer, leading to changes in the molecular structure [18]. It has been demonstrated that the physical characteristics of plastics can undergo substantial changes as a consequence of chain scission or chain growth [19]. In the case of conventional polymers, the influence of multiple recycling cycles has been the subject of extensive analysis. Ben Amor et al. [20] examined the effect of repeated recycling cycles on the properties of polyamide 6 (PA6) and polyamide 66 (PA66). After six cycles, a decline in mechanical properties was observed, with a 36% decrease in the tensile modulus and a 14% decrease in the tensile strength for PA6. In the case of PA66, the reduction amounted to 34% and 64%, respectively. Both materials exhibited a 50% increase in elongation, while the flexure test results demonstrated similar trends. Furthermore, the recycling process resulted in a reduction in melt viscosity, as evidenced by the increase in the melt flow rate (MFR) values. This decline in melt viscosity is likely attributable to chain scission or hydrolysis. In addition, the recycling process enhanced porosity, adversely affecting the mechanical and physical properties of the material. Similar observations were reported by Langwieser et al. [21] for two types of polyethylene (PE-HD and PE-LD) and two types of polypropylene (PP). Following a series of six recycling cycles, both PE grades exhibited reduced molar mass and a decline in melt flow rates, accompanied by a reduction in stabiliser content, particularly with an increase in the number of recycling cycles. The PP grades exhibited a greater degree of degradation, in terms of reduced stabiliser content, lower molar mass, increased melt flow rate, and embrittlement. For both PE and PP grades, the carbonyl index and crystallinity results did not clearly indicate degradation, but correlations between stabiliser content, molar mass, and melt flow rates suggested that chain scissions were dominant in the PP materials.

A number of studies have been conducted on biopolymers and the alterations in their properties that occur during the process of mechanical recycling. PLA has been the subject of the most extensive research due to its ubiquity in 3D printing applications. Shojaeiarani et al. [22] investigated the molecular structure and mechanical and thermal properties of PLA (2003D, NatureWorks LLC), PLA Blend (Bioflex F2110, FKuR Kunststoff GmbH), potato starch-based polymer (Solanyl C2201 Rodenburg Biopolymers), and poly(3-hydroxybutyrate-co-3-hydroxyvalerate) (PHBV) (Y1000P, TianAn biopolymer) following five extrusion and moulding cycles. The findings indicated that both PHBV and PLA exhibited a substantial decrease in average molecular weight (M.W.) as a result of the recycling process. The dynamic mechanical analysis results demonstrated that the virgin polymers exhibited a higher storage modulus, while the recycled polymers showed a more viscoelastic behaviour. Solanyl demonstrated superior thermal processability but inferior recyclability in comparison to the other biopolymers under investigation. The maximum flexural strength of all polymers, with the exception of Solanyl, underwent a decline in response to the number of extrusion cycles. Similarly, Gonçalves et al. [23] analysed the changes in the properties of PLA filaments after five extrusion cycles. The findings demonstrated that PLA has good potential for being mechanically recycled with minimal degradation. After five extrusion cycles, a 30% reduction in M.W. was observed under humid conditions. The presence of moisture has been shown to induce severe degradation by hydrolysis; conversely, dry samples undergo mainly thermal degradation. Whilst discolouration may render the material unsuitable for reuse in certain applications, the overall thermal properties of PLA remain stable.

**Figure 1:**
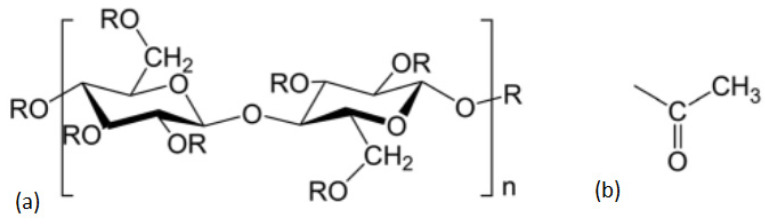
(**a**) General structure of cellulose ester and (**b**) acetyl group as a substitute for the residual group in the general structure of cellulose ester [26].

To the authors’ knowledge, the mechanical recyclability of CA has not yet been investigated. CA is a subgroup of cellulose esters and is considered a cellulose derivative. Cellulose is the most prevalent natural polymer and is mainly derived from wood. The process of chemically modifying cellulose to form a cellulose ester renders it thermoplastic and thus formable [24]. CA is currently employed in a variety of applications, including, but not limited to, photographic films, cigarette filters, ribbons, and labels, as well as textiles and clothing [25]. The residual groups of the cellulose ester may vary depending on the type of cellulose derivative. Figure 1a depicts the general structural formula of cellulose ester. CA is characterised either by the presence of a hydrogen atom or an acetyl group as a residual group, as indicated in Figure 1b. As illustrated in Figure 1a, three residual groups are attached to each glucose building block. This enables the creation of a range of substitution degrees, which are denoted by the average degree of substitution. Consequently, if all three OR groups are substituted by acetyl groups, for instance, this would be designated as DS = 3. For CA, a typical degree of substitution is DS ~ 2.5 [26].

The present study focuses on the end-of-life management of a commercially available CA-based blend (ARBOBLEND^®^ 4655V) through mechanical recycling, which has the benefit of reducing landfill disposal and conserving new resources. The authors pursue the idea that biopolymers should be collected and mechanically recycled alongside conventional waste streams. Only if biopolymers can no longer be reused or mechanically recycled should they be biologically recycled by degradation. Hence, the recyclability of this CA-based blend was examined by subjecting it to four recycling passes of repeated shredding and injection moulding. The mechanical, thermal, and rheological properties were studied after each cycle to assess the performance of the recycled ARBOBLEND^®^ 4655V.

Cellulose acetate (CA) has gained increasing attention as a biobased thermoplastic for technical applications because it combines renewable feedstock, good mechanical performance, and established processing routes (e.g., injection moulding, extrusion) with a relatively high glass transition temperature and chemical resistance. At the same time, CA is known to be thermally sensitive, as its processing window lies close to its onset of thermal degradation and deacetylation [27]. In practical terms, this means that CA-based compounds are particularly susceptible to property changes during melt processing and repeated reprocessing.

In the literature, the durability of CA and other cellulose derivatives has mainly been studied with respect to hydrolytic stability, enzymatic and microbial degradation, photooxidation, and weathering, as well as thermo-oxidative ageing [28]. These studies typically focus on changes in molar mass, degree of substitution, crystallinity, and mechanical properties after controlled exposure in aqueous, soil, compost, or accelerated ageing environments. In contrast, there is only limited information on how repeated melt processing (mechanical recycling) affects the structure–property relationships of CA-based materials under industrially relevant conditions.

In this context, the choice of ARBOBLEND^®^ 4655V as a cellulose–acetate-based model system is motivated by its status as a commercially available injection-mouldable compound already used in technical applications. The processing temperatures investigated in this work (170 °C, 190 °C, and 210 °C) were selected to span the recommended temperature window for ARBOBLEND^®^ V grades while deliberately bracketing the lower, intermediate, and upper limits of the practical processing range. This allows the influence of ‘mild’ (170 °C), ‘typical’ (190 °C), and ‘critical’ (210 °C) processing conditions on the recyclability to be assessed.

The combination of rheological, thermal, and mechanical testing was chosen to capture different levels of the structure–property relationship: (1) rotational rheometry and oscillatory measurements provide rheology-based indications of changes in average molecular weight and molecular-weight distribution during recycling; (2) differential scanning calorimetry reveals associated changes in crystallisation and melting behaviour; and (3) tensile and impact tests quantify the resulting performance in terms of stiffness, strength, and toughness. Together, these methods enable a mechanistic assessment of how processing temperature and recycling cycle number affect the durability of this cellulose–acetate-based blend under repeated mechanical recycling.

## 2. Materials and Methods

### 2.1. Materials

The natural-coloured ARBOBLEND^®^ 4655V was supplied by Tecnaro GmbH (Ilsfeld, Germany) in the form of 3 mm pellets. According to the supplier, ARBOBLEND^®^ 4655V is a cellulose–acetate-based biopolymer blend composed primarily of CA with an elastic modulus of 1400 MPa, a tensile strength of 24 MPa, and an elongation at break of 12%, as per the material’s technical datasheet. The recommended processing temperature for injection moulding is specified for the entire range of ARBOBLEND^®^ V products and is proposed to fall between 170 °C and 210 °C. The supplier did not disclose any information with regard to the further composition of the blend. However, depending on the formulation, ARBOBLEND^®^ materials generally consist of biopolymers such as PHA, PLA, polycaprolactone (PCL), polyester (e.g., bio-PET), starch, bio-PE, bio-PA, lignin, natural resins, waxes, oils, natural fatty acids, cellulose, biological additives, and natural reinforcing fibres. Previous research has demonstrated the biodegradability of ARBOBLEND^®^ V plastics [29].

### 2.2. Sample Preparation

The ARBOBLEND^®^ 4655V granules were dried at 50 °C for 4 h in a dryer type DR 204 MT manufactured by Bierther Systemtechnik (Bad Kreuznach, Germany) before being further injection moulded into Type A1 tensile specimens according to DIN EN ISO 527-2 [30], using a Demag System 80/420-430 injection moulding machine (Schwaig bei Nürnberg, Germany) equipped with a film-gate double-cold-runner mould. The material was injection moulded at three distinct temperatures of 170 °C, 190 °C, and 210 °C to study the effect of temperature on the property profile of the CA-based blend. The detailed injection moulding parameters are given in Table 1.

To investigate the effect of multiple recycling passes on the processability and properties of ARBOBLEND^®^ 4655V, four recycling runs were conducted at each of the aforementioned temperatures. Virgin samples (R0) were left to cool for 30 min after injection moulding and were further shredded using a Rapid Granulator 150 series manufactured by Rapid Granulator AB (Bredaryd, Sweden). These mills provide a re-grind of 3–5 mm that can be directly fed back into the hopper of the injection moulding machine [31]. The shredded and reprocessed material, termed ‘recycled’, was then used to produce new tensile samples after drying according to the aforementioned conditions. This process was repeated four times, rendering test specimens labelled as R1, R2, R3, and R4, respectively.

### 2.3. Characterisation

Virgin and recycled samples were examined with respect to their chemical structure, as well as their mechanical, rheological, and thermal behaviour. Unless otherwise stated, experimental results are reported as mean values ± standard deviation (mean ± SD) based on at least five replicates for mechanical tests and three replicates for rheological and thermal measurements.

#### 2.3.1. Rotational Rheometry

**Figure 2:**
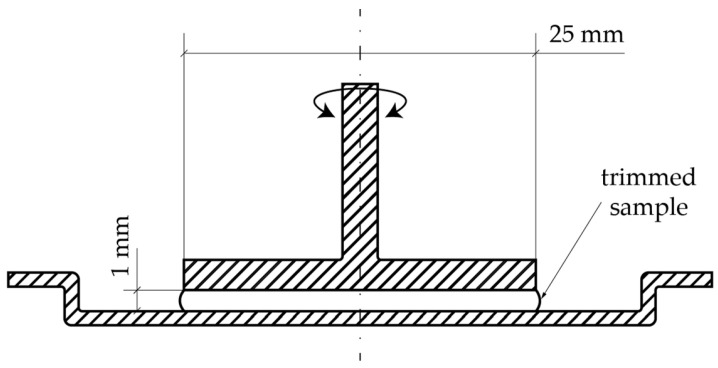
Schematic illustration of the rheological plate–plate setup used.

The viscosity profile of virgin pellets and shredded recycling materials was investigated using a Modular Compact Rheometer MCR 302 from Anton Paar (Ostfildern-Scharnhausen, Germany) in rotational mode and a plate–plate setup with a plate diameter of 25 mm and a gap distance of 1 mm (Figure 2) at a constant temperature of 160 °C and a shear ramp from 0.01 s^−1^ to 1000 s^−1^. This setup was chosen because it is well-suited for highly viscous polymer melts and enables reliable measurements of heterogeneous samples such as shredded recyclate without blockage or excessive edge effects. Three replicate measurements were carried out for each material. CA is commonly known to be soluble in acetone. However, the ARBOBLEND^®^ 4655V could not be dissolved in acetone or in other common solvents such as chloroform. Consequently, it was not possible to quantify the molecular weight (M.W.) and the molecular weight distribution (MWD) by gel permeation chromatography or to determine a solution viscosity number. Therefore, rheological measurements were used as qualitative indicators in an attempt to determine the relative changes in rheology-based M.W. and MWD as a function of recycling cycles and processing temperature. An oscillation test was carried out in the frequency range of 1 Hz to 100 Hz at a constant temperature of 160 °C and a constant shear deformation of 0.1%. The main objective of the oscillation test was to evaluate the storage and loss moduli. Based on shifts in the position of the rheological crossover point, qualitative statements could be made about possible changes in the average M.W. or MWD of the ARBOBLEND^®^ 4655V and its recyclates, without implying direct quantitative molecular weight determination.

#### 2.3.2. Infrared Spectrometry

Infrared (IR) spectra of the virgin and recycled ARBOBLEND^®^ 4655V were measured at each processing temperature with a Shimadzu IRTracer-100 (Kyoto, Japan) in the mid-infrared range using an attenuated total reflection (ATR) unit equipped with a diamond puck in reflectance mode. The measurements were performed using a resolution of 4 cm^−1^, conducting 16 scans per spectrum.

#### 2.3.3. Differential Scanning Calorimetry

Differential Scanning Calorimetry (DSC) was used to evaluate the glass transition (T_g_) and melting temperature (T_m_) of the virgin and recycled CA-based blend, processed at different temperatures. The Mettler Toledo calorimeter DSC 3+ (Columbus, OH, USA) was used. Samples of approximately 5 mg were punched from the shoulder area of untested tensile test specimens and introduced into a 0.4 µL aluminium crucible. A preliminary investigation was conducted over the temperature range of 0 °C to 400 °C in order to ascertain the temperature range of interest for further investigation. Each sample was then subjected to a temperature programme consisting of a first heating cycle from 0 °C to 160 °C at a rate of 20 K/min, followed by cooling back down to 0 °C at the same rate, and finally a second heating under the same conditions as the first. The temperature was held isothermally for 2 min between successive steps. The tests were carried out in a nitrogen atmosphere at a purge rate of 60 mL/min.

#### 2.3.4. Tensile Testing

Tensile tests were performed according to DIN EN ISO 527-1 [32] using a Shimadzu Autograph AG-C plus universal testing machine (Kyoto, Japan) using a 10 kN load cell and an optical extensometer (TRViewX). Five tensile specimens each of virgin and recycled material were tested at room temperature. A preload of 10 N was applied to the specimen at the start of the test, followed by continuous loading at a crosshead speed of 1 mm/min up to 0.25% strain to determine the tensile modulus in this range. The test speed was then increased to 50 mm/min until sample fracture. For each specimen, the stress–strain curve was recorded and further evaluated to determine tensile modulus (E), tensile strength (σ), and strain (ε).

#### 2.3.5. Charpy Impact Testing

Impact bending tests were performed on a Zwick pendulum impact tester in accordance with DIN EN ISO 179-1/1eU [33]. Unnotched specimens of each virgin and recycled material were cut from the gauge length of the type A1 tensile test specimens, injection moulded in accordance with DIN EN ISO 3167 [34]. The samples were tested edgewise at room temperature, using an impact pendulum with a working capacity of 7.5 J. The impact strength (a_cU_) of each material was calculated as the average of five replicates.

#### 2.3.6. Statistical Analysis

Separate one-way ANOVA and Tukey HSD post hoc analyses were conducted using JMP^®^ Pro 17.0.0 to compare the effects of processing temperature and number of recycling cycles on the rheological, thermal, and mechanical properties of ARBOBLEND^®^ 4655V. Each factor (processing temperature, recycling cycle) was analysed individually in separate one-way designs. Statistical significance was assessed at a significance level of α = 0.05. Effect sizes were evaluated based on relative changes in the corresponding mean values (mean ± SD).

## 3. Results and Discussion

### 3.1. Sample Preparation

**Figure 3:**
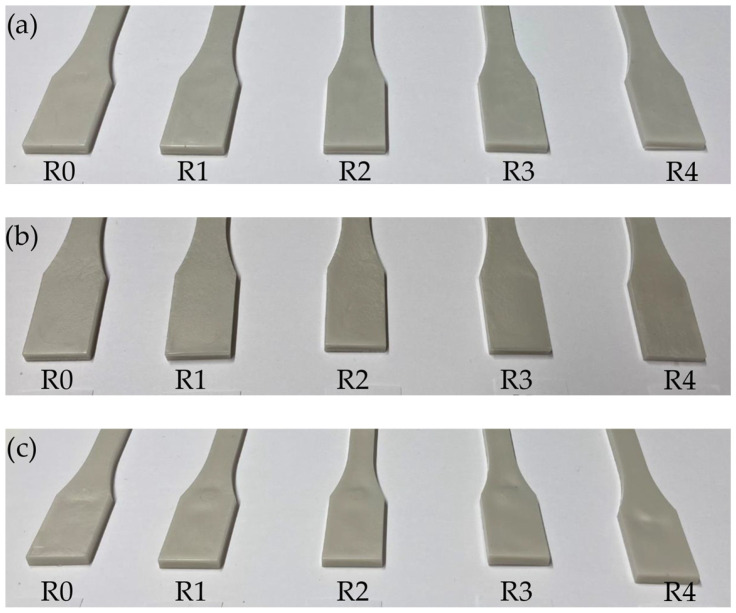
Samples of the different recycling stages R0 to R4 injection moulded from ARBOBLEND^®^ 4655V at 170 °C (**a**), 190 °C (**b**), and 210 °C (**c**).

Figure 3 shows the injection-moulded samples made from virgin ARBOBLEND^®^ 4655V and the four recycling stages R1 to R4. The surface quality of the recycled samples decreased with increasing cycle number, regardless of the moulding temperature. The colour of the samples remained consistent.

### 3.2. Rheological Behaviour

**Figure 4:**
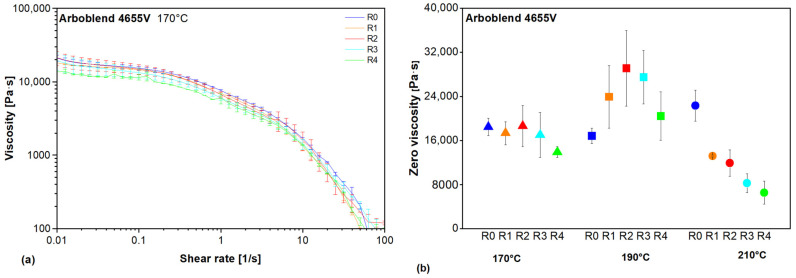
(**a**) Viscosity curve of virgin and recycled ARBOBLEND^®^ 4655V processed at 170 °C, and (**b**) zero-shear viscosity of virgin and recycled ARBOBLEND^®^ 4655V processed at 170 °C, 190 °C, and 210 °C.

The effects of the recycling passes and the processing temperature on the viscosity curve of ARBOBLEND^®^ 4655V were determined using the shear-ramp setup of the rotational rheometry. No significant changes in the overall profile of the viscosity curves were observed, particularly in the region of pseudoplastic transition. Figure 4a shows the viscosity curves of virgin and recycled ARBOBLEND^®^ 4655V processed at 170 °C. The zero-shear viscosity was observed to shift as a result of recycling. Figure 4b presents the average zero-shear viscosity of the virgin and recycled ARBOBLEND^®^ 4655V for all processing temperatures.

The ANOVA confirmed a significant change in the zero-shear viscosity across the recycling passes at all processing temperatures. At 170 °C (F(4, 5) = 8.89, *p*-Value = 0.017), the zero-shear viscosity of R4 is significantly lower compared to all other cycles at this processing temperature, whereas no significant differences in zero-shear viscosity could be detected between R0, R1, R2, and R3. At 190 °C (F(4, 5) = 9.64, *p*-Value = 0.014), the post hoc analyses showed significant differences among R0, R2, and R3, with the zero-shear viscosity of R0 being lower than that of R2 and R3. At 210 °C (F(4, 5) = 8.67, *p*-Value = 0.018), significant differences among R0, R3, and R4 were observed. The zero-shear viscosity of R0 was higher than that of R3 and R4. Overall, there is a tendency for the zero-shear viscosity at 170 °C and 210 °C to decrease with an increasing number of recycling passes, whereas at 190 °C, it initially increased, and then decreased again at R4. However, the measured data showed high scatter, so the trend should be interpreted with caution. This observation is further supported by the DSC results in Section 3.3.

**Figure 5:**
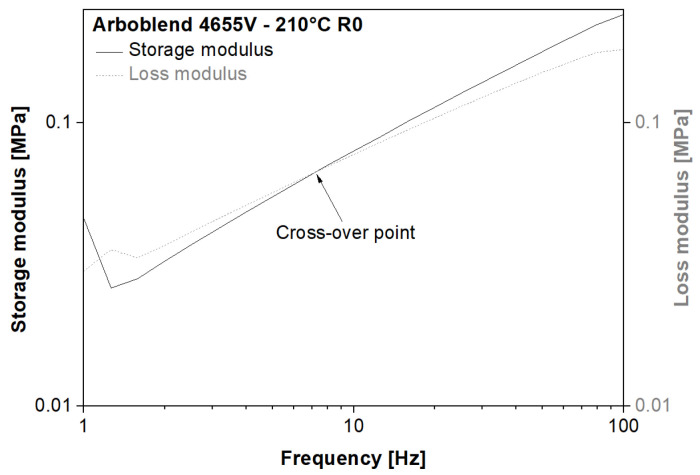
Storage and loss modulus of R0 ARBOBLEND^®^ 4655V, processed at 210 °C as a function of the frequency.

**Figure 6:**
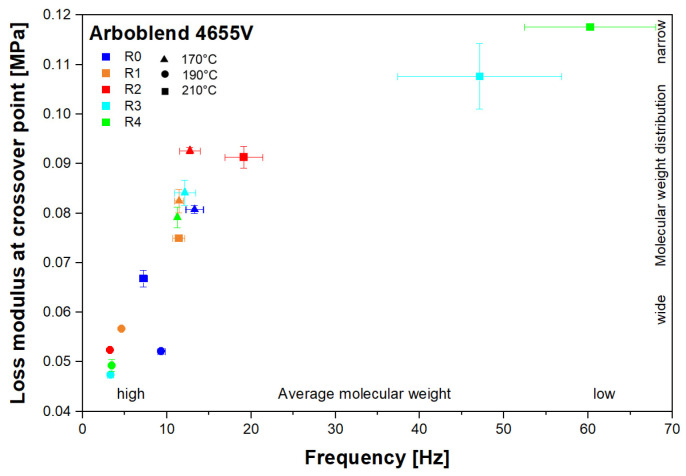
Crossover point of the storage and loss moduli of ARBOBLEND^®^ 4655V measured with rotational rheometry in oscillatory mode for the different recycling cycles at varying processing temperatures of 170 °C, 190 °C, and 210 °C.

Generally, a change in zero-shear viscosity can indicate a change in average M.W. [35]. In this study, rheology-based indications of changes in the average M.W. and the MWD were identified by analysing the position of the crossover point of the storage and loss modulus curves as a function of frequency. Figure 5 shows such curves and the resulting crossover point for R0 processed at 210 °C. Figure 6 compares the crossover points of all recycling runs at the different processing temperatures under investigation. The vertical shift of the crossover point correlates with a change in the MWD, while the horizontal shift provides information on the change in the average M.W. [35].

It is evident that at 170 °C (Figure 6), the average M.W. is not significantly affected by multiple processing cycles, as also confirmed by the ANOVA. However, the vertical shift of the crossover point, which correlates with a change in the MWD of cellulose acetate, appears to be significant (F(4, 5) = 7.97, *p*-Value = 0.021). The post hoc analysis showed a significant upward shift in the crossover point of R2 with respect to R0 and R4, and consequently a significantly narrower MWD. A narrower MWD indicates decreased polydispersity (polydispersity index PI = M_w_/M_n_, where M_w_ is the weight-average molecular weight and M_n_ is the number-average molecular weight), i.e., there is a smaller variation in the lengths of the polymer chains. During polymer processing, chain scission can occur as a result of mechanical shear, causing the polymer chains to break into smaller fragments. This process increases the number of shorter chains. Assuming that the weight-average molecular weight remains unchanged, the number-average molecular weight decreases, thereby broadening the MWD [36].

Increasing the processing temperature to 190 °C causes significant changes in the position of the crossover point (Figure 6). The post hoc analysis showed a significant shift of the crossover point to the left for all recycling passes with respect to R0, which can be interpreted as a rheological indication of an increased weight-average M.W. Simultaneously, a significant downward shift of the crossover point is observed for R1 to R4, indicating a broader MWD as a result of increasing the number of recycling passes. At 190 °C, the broader molecular weight distribution can be attributed to two opposite processes: (1) chain scission due to mechanical shearing, leading to a decrease in the number-average molecular weight M_n_, and (2) acetylation, leading to increased branching and thus an increase in the weight-average molecular weight M_w_. The experiments at 190 °C were repeated and yielded consistent rheological trends, supporting the indication of an increased average M.W. at this processing temperature.

At 210 °C, the ANOVA also indicated significant changes in horizontal (F(4, 5) = 17.016, *p*-Value = 0.00409) and vertical (F(4, 5) = 44.207, *p*-Value = 0.0004) shifts. The magnitude of the shifts at 210 °C is much greater than the changes observed at 170 °C and 190 °C (Figure 6). During melt processing, CA is prone to thermal degradation due to its melting temperature being close to its decomposition temperature. This degradation can lead to a reduction in the degree of acetylation, as acetyl groups are lost during the process [37]. This explains the degradation in terms of both chain scission and deacetylation, leading to an overall rheology-based indication of a reduction in the average M.W. and a narrower MWD.

These results are consistent with the observation that the zero-shear viscosity of the CA-based blend increases when recycled at 190 °C, whereas the viscosity decreases when the material is repeatedly recycled at 210 °C (Figure 4).

In addition to changes in viscosity and rheology-based indications of changes in average M.W. and MWD as a result of multiple recycling passes, the ANOVA further showed a significant dependence on processing temperature. For example, for R0, a vertical and horizontal shift of the crossover point could be observed, where F(2.3) = 165.69, *p*-Value = 0.0008 for the vertical shift, and F(2.3) = 20.69, *p*-Value = 0.0175 for the horizontal shift. As evidenced by the post hoc analysis, all three temperatures have significant differences in the vertical shifts of R0. Rheological analysis suggests that the MWD of R0 at 190 °C was the widest, while it was the narrowest at 170 °C. There was also a significant difference in the horizontal shift in the crossover point between 170 °C and 210 °C. R0 processed at 210 °C indicates a higher average M.W. than R0 processed at 170 °C. Thus, the processing temperature has a significant influence on the rheologically inferred average M.W. and the MWD.

### 3.3. Chemical Structure

**Figure 7:**
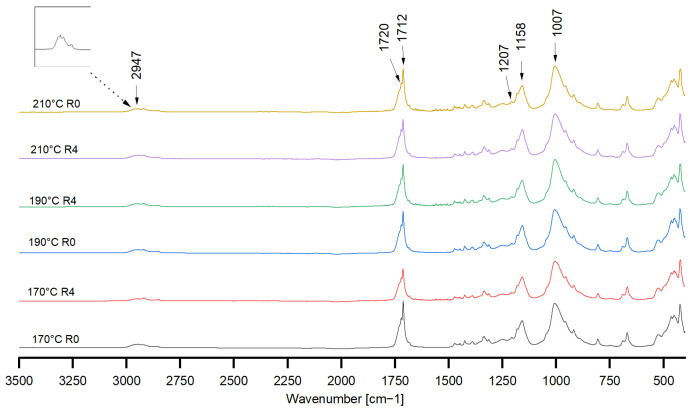
Infrared spectra of virgin (R0) and recycled (R4) ARBOBLEND^®^ 4655V processed at different temperatures.

The spectra of the R0 and R4 samples are shown in Figure 7 as an example. No significant alterations in the spectra were observed across the varying processing temperatures or the recycling cycles.

The bands specific to the acetyl structure of CA are identified at approximately 1720 cm^−1^ (C=O stretching) and 1207 cm^−1^ (C-O stretching with acetate residue), in addition to 1007 cm^−1^ (C-O stretching) [38]. Furthermore, the spectrum displays pronounced bands at 1712 cm^−1^ and 1158 cm^−1^, which, upon examination of the extant literature, do not appear to be directly attributable to CA. These features may originate from further constituents of the commercial blend. First thoughts were oriented to polybutylene succinate (PBS), which is often compounded with CA to improve processability [39,40,41]. However, based on FTIR alone, the presence of PBS in ARBOBLEND^®^ 4655V cannot be confirmed unambiguously.

A comparison of the spectrum with the IR spectra of PBS clearly demonstrates the difficulty of determining with certainty that PBS is the other component of the blend, as it is evident that both polymers possess ester structures, resulting in overlap of specific peaks. In a publication by Hu et al. [41], PBS/CA blends were analysed using FTIR. The findings demonstrated that, with a mixing ratio of up to 10% CA to PBS, there were visible changes in the spectrum when compared to the pure PBS spectrum. The specific peak for PBS at 1715 cm^−1^, indicative of the -COO- bond in the ester group, and the peak at 1730 cm^−1^ characteristic of the carbonyl group of CA, was shifted and superimposed on the double-peak at 1720 cm^−1^ and 1712 cm^−1^ as a result of blending [42]. This would be consistent with the recorded spectrum in Figure 7, which shows strong peaks at 1712 cm^−1^ and 1720 cm^−1^. A further qualitative indication that PBS might be present in the polymer blend under investigation is the more pronounced occurrence of aliphatic C-H stretching bands at 2800–3000 cm^−1^ [43]. Nevertheless, these FTIR observations can only suggest, but not definitely prove, the presence of PBS. Additional chemical analysis would be required for unambiguous identification. However, the results of the DSC analysis further support the interpretation that ARBOBLEND^®^ 4655V behaves like a CA-based blend containing a low-melting-point polyester component, as discussed in the next section.

### 3.4. Thermal Behaviour

**Figure 8:**
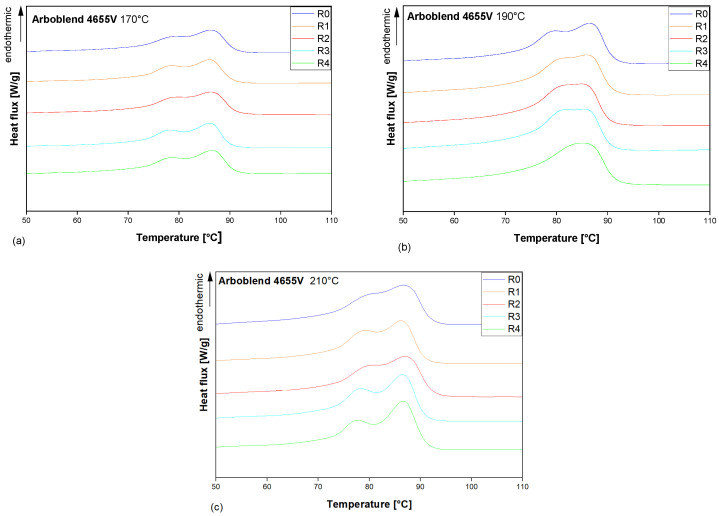
Melting region recorded during the second heating of the DSC for virgin and recycled ARBOBLEND^®^ 4655V processed at different temperatures: (**a**) 170 °C, (**b**) 190 °C, and (**c**) 210 °C.

As illustrated in Figure 8, the melting peaks captured during the second heating of the DSC for R0 to R4 ARBOBLEND^®^ 4655V processed at 170 °C, 190 °C, and 210 °C are presented. In the majority of cases, a double melting peak could be observed within the temperature range from 70 °C to 90 °C. Table 2 summarises the onset and offset temperatures of the double peak, the overall melting enthalpy, and the mean temperature of each peak maximum for all cases. The ANOVA shows no significant changes in the melting peaks or total melting enthalpy across the different recycling cycles within all three processing temperatures.

The melting peak temperature is surprisingly low and does not align with the findings in the literature, which assert that the melting temperature of neat CA typically falls within the range of 150 °C to 240 °C [44,45,46]. Furthermore, the present DSC analysis does not directly reflect the manufacturer’s recommendation for processing the material within the range of 170 °C to 210 °C. This might be attributed to the composition of the ARBOBLEND^®^ 4655V blend, as opposed to a pure CA formulation. The low-temperature double-melting peak is consistent with the presence of a low-melting polyester component. In the literature, PBS is recognised for its melting point of approximately 100 °C [47], which can be reduced even further by copolymerisation [48]. According to the literature, PBS exhibits a complex melting behaviour, which is characterised by reorganisation of the crystalline structures with different thermal stabilities. As a result, PBS is known to exhibit bimodal peaks [49]. In ARBOBLEND^®^ 4655V, the observed double peak between 70 °C and 90 °C therefore suggests PBS-like behaviour, but, in line with the FTIR results, does not constitute definitive proof of PBS as a specific blend component. From a morphological point of view, the coexistence of low- and high-melting-point crystalline populations indicates that the polyester phase in the blend crystallises into lamellae with different thicknesses and degrees of perfection. Recycling-induced changes in molecular-weight distribution and chain architecture (e.g., increased branching at 190 °C or chain scission at 210 °C) modify the crystallisation kinetics and lamellar stability. This provides a mechanistic link between the rheologically inferred changes in MWD and the evolution from a broad monomodal peak at 190 °C to a sharper bimodal melting behaviour at 210 °C.

The shape of the melting peak strongly depends on the processing temperatures. The double peak is clearly evident for the ARBOBLEND^®^ 4655V processed at both 170 °C and 210 °C (see Figure 8a and Figure 8c, respectively). However, at 190 °C, the peak evolves to a single broad mono-modal peak. The rheological tests showed that for the blend processed at 190 °C, the rheology-based average M.W. is higher and the MWD broader than at other processing temperatures, especially after repeated recycling. Svintradze et al. [50] posit that an increase in M.W. is associated with the formation of more thermally stable crystal structures. It has been further demonstrated that the broader MWD results in an increase in the variety of different crystalline structures, with the melting peaks of these structures merging into one another.

At 210 °C, the effect is different. With an increasing number of recycling passes, the melting peaks evolve into a more pronounced shape with sharper individual peaks in the bimodal melting peak (Figure 8c). This can be interpreted in light of the changes within the MWD. This correlates with the general statement [51] that a narrower MWD results in a more defined transition area compared to a broader MWD. Consequently, the narrower the MWD, the narrower and sharper the double peak (Figure 8c). Conversely, the samples R1, R3, and R4 processed at 190 °C, which exhibit the broadest MWD according to rheological analysis, show a broad, essentially non-bimodal peak (Figure 8b).

**Figure 9:**
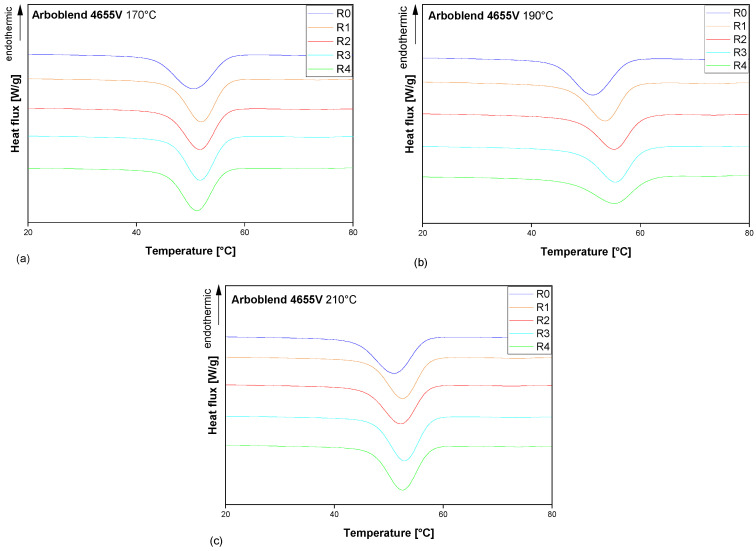
Crystallisation region recorded during the second heating of the DSC for virgin and recycled ARBOBLEND^®^ 4655V processed at different temperatures: (**a**) 170 °C, (**b**) 190 °C, and (**c**) 210 °C.

Figure 9 illustrates the cut-out of the crystallisation reaction in the first DSC cooling stage, carried out on virgin and recycled ARBOBLEND^®^ 4655V, processed at the three temperatures under investigation. Table 3 shows the mean values of the identified peaks, the onset, the offset, and the crystallisation enthalpy. At 170 °C, the ANOVA showed no significant differences in the crystallisation peak, crystallisation onset, crystallisation offset, or the crystallisation enthalpy.

At 190 °C, the peak shape changes significantly across all recycling passes. According to the ANOVA the crystallisation onset (F(4, 5) = 238.55, *p*-Value = 0.00001), crystallisation offset (F(4, 5) = 10.37, *p*-Value = 0.01225), crystallisation peak (F(4, 5) = 17.41, *p*-Value = 0.00387), and the crystallisation enthalpy (F(4, 5) = 7.25, *p*-Value = 0.02595) vary significantly. The crystallisation peak is shifted towards higher temperatures over the recycling passes. According to [51], a shift of the crystallisation peak in the direction of higher temperatures corresponds to molecular chain degradation. At first glance, this statement does not correlate with the results of the rheological tests. However, the post hoc analysis showed significant differences between the crystallisation onset of the various recycling passes. According to [52], a shift towards higher temperatures of the onset implies an earlier crystallisation, due to nucleation encouraged by the existing short-chain molecules, acting as nucleating agents [53]. Such short-chain molecules are a result of the broader MWD [54], as a result of recycling at 190 °C. However, the average M.W. indicated by rheology generally leads to a lower crystallinity. This is supported by the reduced crystallisation enthalpy between R0 and R4. This behaviour is consistent with the classical view that higher average M.W. and branching hinder chain mobility and slow down crystal growth, while the presence of shorter chains promotes nucleation but contributes less to overall crystallinity. The combined effect at 190 °C is therefore an earlier onset of crystallisation, driven by short-chain nucleation, but a lower overall crystallisation enthalpy due to the reduced ability of the branched, higher-molecular-weight chains to organise into well-defined lamellae.

At 210 °C, the ANOVA indicated that the crystallisation onset (F(4, 5) = 22.63, *p*-Value = 0.0021) and the crystallisation offset (F(4, 5) = 16.95, *p*-Value = 0.0041) slightly changed between R0 and the other recycling cycles. The crystallisation enthalpy (F(4, 5) = 7.25, *p*-Value = 0.02595) significantly depends on the number of recycling passes. Again, this correlates with the rheological results, which showed a strong narrowing of the MWD and, in turn, the associated higher crystallinity. The crystallisation peak (F(4, 5) = 9.61, *p*-Value = 0.0145) of R0 is significantly shifted towards higher temperatures compared to all other recycling passes. In this case, the hypothesis that the crystallisation peak is shifted towards higher temperatures as the chains become shorter [51] is applicable.

### 3.5. Tensile Testing

**Figure 10:**
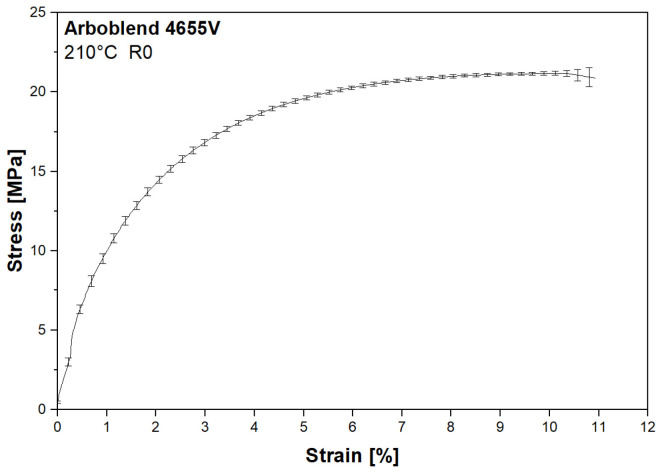
Stress–strain curve of the virgin ARBOBLEND^®^ 4655V processed at 210 °C.

Figure 10 shows an exemplary stress–strain curve recorded during tensile testing of ARBOBLEND^®^ 4655V after first processing (R0) at 210 °C. The other temperatures under investigation and the further recycling passes exhibited similar stress–strain profiles.

**Figure 11:**
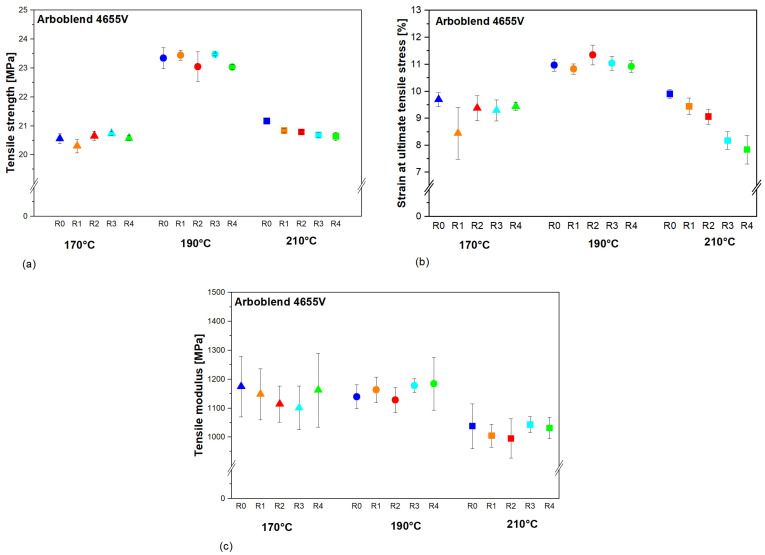
(**a**) Tensile strength, (**b**) elongation at ultimate tensile stress, and (**c**) tensile modulus for the virgin and recycled ARBOBLEND^®^ 4655V processed at 170 °C, 190 °C, and 210 °C. The colour code denotes the recycling step, whereas the shape code reflects the processing temperature.

Figure 11a shows the mean tensile strength for the R0-R4 ARBOBLEND^®^ 4655V processed at 170 °C, 190 °C, and 210 °C. At 170 °C and 190 °C, no significant changes in the tensile strength as a function of recycling passes could be detected. At 210 °C, the ANOVA and subsequent post hoc tests showed a significant difference between the tensile strength of R0 and the other recycling passes, R1–R4. In practical terms, the tensile strength decreased from 21.2 ± 0.08 MPa at R0 to 20.6 ± 0.15 MPa at R4, corresponding to a relative reduction of approximately 3%, indicating a slight but statistically significant loss in strength with increasing recycling runs. Moreover, the tensile strength of R0 was found to be significantly dependent on the processing temperature (F(2, 9) = 174.28, *p*-Value ˂ 0.0001), where the tensile strength is minimum at 170 °C (20.56 ± 0.17 MPa) and maximum at 190 °C (23.34 ± 0.36 MPa). This corresponds to an increase in tensile strength of approximately 10–14% when the processing temperature is raised from 170 °C to 190 °C, representing a clearly larger effect size for processing temperature than for repeated recycling. It is worth noting that the increase in the processing temperature from 170 °C to 190 °C resulted in an increase in tensile strength of approximately 10%, which is much higher than the influence of repeated recycling passes, where the maximum variation of approximately 2% was observed at 210 °C in the case of R4 with respect to R0. Thus, although the influence of recycling passes at 210 °C is statistically significant, the associated effect size on tensile strength is small compared to the pronounced effect of processing temperature. This indicates that the recycling temperature has a greater impact on tensile strength than the number of recycling passes and must therefore be carefully selected. The strength of polymers is generally affected by their M.W. and MWD [19]. The analysis of rheology-based changes in M.W., as mentioned above, revealed an increased average M.W. for the ARBOBLEND^®^ 4655V recycled at 190 °C, which can be correlated with the increased tensile strength. A further temperature increase to 210 °C could be associated with more severe degradation effects, leading to decreased M.W. and, in turn, to a reduced tensile strength. On a microstructural level, the moderate increase in rheology-based average M.W. and branching at 190 °C appears to enhance load transfer between chains and stabilise the crystalline phase, which explains the higher tensile strength at this temperature. At 210 °C, however, chain scission and deacetylation dominate, reducing the effective chain length and weakening intermolecular entanglements, so that the material can no longer sustain the same stress level despite a partially higher crystallinity.

Figure 11b shows the strain at maximum tensile stress (ε_u_) for all recycling passes at 170 °C, 190 °C, and 210 °C. According to the ANOVA, there was no significant effect of the repeated recycling on the strain for the ARBOBLEND^®^ 4655V recycled at 170 °C. However, at 190 °C, the post hoc analysis showed a significant increase of 0.5% in the strain from R1 to R2. This corresponds to a very small effect size, indicating only a minor improvement in ductility at this temperature. At 210 °C, ε_u_ significantly decreased with each recycling run (F(4, 20) = 32.02, *p*-Value ˂ 0.0001). With each run, ε_u_ decreased by approximately 5%; the third recycling cycle R3 was even associated with a 10% decrease in ε_u_ with respect to the value measured for R2. Overall, this amounts to a marked cumulative reduction in strain at maximum tensile stress over multiple recycling passes at 210 °C, reflecting a substantial effect size on ductility. Generally, the maximum ε_u_ was detected for recycled ARBOBLEND^®^ 4655V processed at 190 °C. This could be attributed to the increased MWD. First, short chains may act as lubricants, facilitating the sliding of the longer chains. Second, the rheologically supported increased weight-average molecular weight is a result of increased branching, which widens the distance between the chains and results in decreased secondary bonding between the molecular chains. Both aspects lead to an increase in tensile strain, as depicted for the recycled ARBOBLEND^®^ 4655V at 190 °C. Mechanistically, this combination of a broader MWD with shorter, more mobile chains and branched, longer load-bearing chains facilitates local plastic deformation and delays crack initiation, which is reflected in the higher strain at maximum tensile stress observed at 190 °C. In this context, the effect size of processing temperature on ε_u_ is clearly larger than the effect of repeated recycling at 170 °C, whereas at 210 °C, the number of recycling passes has a strong negative effect on ε_u_.

Figure 11c presents the tensile modulus of ARBOBLEND^®^ 4655V for all recycling passes at 170 °C, 190 °C, and 210 °C. The measurement of the tensile modulus was accompanied by large scattering, and no significant dependence on either the number of recycling cycles or the recycling temperature was found. Generally, the scatter of the results at 170 °C appears to be greater for all recycling cycles R0–R4. At 170 °C and 190 °C, the single greatest standard deviation was observed for cycle R4, whereas at 210 °C, it was observed for R0. At the same time, for each temperature range, overlapping data points exist, which may also point to deviations during the testing procedure. Consequently, even where slight differences in mean modulus are visible, the associated effect sizes are small compared to the variability of the data and must be interpreted with caution, and no robust trend with respect to recycling cycle or processing temperature can be established.

### 3.6. Charpy Impact Testing

Table 4 shows the impact strength of the virgin and recycled ARBOBLEND^®^ 4655V processed at 170 °C, 190 °C, and 210 °C. It is to be noted that no fracture was achieved for any of the samples processed at 190 °C. At 170 °C and 210 °C, only a few samples exhibited failure. The ANOVA showed no significant changes in impact strength at 170 °C as a function of the recycling passes. At 210 °C, there are significant changes in the impact strength (F(2, 10) = 12.99, *p*-Value = 0.0017), where the post hoc analysis showed significant differences between R4 on the one hand and R2 and R3 on the other hand. The impact strength of R4 is approximately 36% lower than that of R2, indicating a large effect size of repeated recycling at 210 °C on impact performance. Generally, the impact strength of a polymer decreases with decreasing chain length [19]. This correlates with the aforementioned results, where the highest rheology-based average M.W. was detected for all the recycling cycles processed at 190 °C. In this case, no fracture was observed upon impact loading. At 210 °C, the polymer seems to degrade most, leading to a significantly lower rheology-based average M.W. and thus reduced impact strength. In terms of fracture mechanics, the absence of impact fracture at 190 °C suggests that sufficient energy can be dissipated through shear yielding and microplasticity in the ductile polyester-rich phase, whereas at 210 °C, the shortened, degraded chains and reduced entanglement density promote brittle crack propagation and a pronounced drop in impact strength.

## 4. Conclusions

In this study, the properties of ARBOBLEND^®^ 4655V were analysed after repeated mechanical recycling at three different temperatures. The IR results demonstrated that there were no significant changes in the material across the recycling cycles at all processing temperatures. However, based on FTIR and DSC, it was suggested that the commercial material behaves as a cellulose–acetate-based blend containing a low-melting polyester phase. This has been tentatively discussed in terms of PBS-like features, but the exact blend composition remains undisclosed and cannot be confirmed within the scope of this study.

Thermal and rheological investigations indicated that the tendencies of the property changes were different at each processing temperature. In the context of the present study, the lowest processing temperature (170 °C) was observed to have no significant impact on zero-shear viscosity or thermal properties, including the melting peak and crystallisation, and therefore did not provide clear rheological indications of changes in the average molecular weight or molecular weight distribution. The mechanical properties remained constant throughout the recycling cycles.

Within the average processing temperature (190 °C), rheological measurements indicated a slight increase in the rheology-based average molecular weight over the recycling passes and a shift in the rheological crossover, which can be interpreted as a possible broadening of the molecular weight distribution. It is evident that this has a direct impact on the shape of the melting peak. The double peak, which was evident at all processing temperatures, underwent a transition to a single peak at 190 °C during multiple recycling cycles. Furthermore, the crystallisation behaviour exhibited alterations across the recycling runs. The crystallisation temperature exhibited a shift towards higher temperatures, accompanied by a decline in the degree of crystallisation. However, these minor changes had no significant effect on the mechanical properties of ARBOBLEND^®^ 4655V, and the associated effect sizes on tensile strength and strain remained small.

At the highest processing temperature under investigation (210 °C), a decrease in zero-shear viscosity and a shift in the rheological crossover across the recycling passes suggest a reduction in the rheology-based average molecular weight and a gradual narrowing of the molecular weight distribution, without providing direct molecular weight quantification. In contrast to the observations made at a processing temperature of 190 °C, the double melting peak at 210 °C was much more pronounced across the recycling passes. The crystallisation temperature shifted towards higher temperatures, accompanied by an increased degree of crystallisation. These changes were more pronounced, which had an impact on the mechanical properties of ARBOBLEND^®^ 4655V. The impact strength, strain at ultimate tensile stress, and strain at break decreased with each recycling run, indicating substantial effect sizes, particularly at 210 °C. The tensile strength, modulus of elasticity, and breaking stress remained largely stable.

The above-mentioned observations support the hypothesis that processing temperature has a major influence on the behaviour of ARBOBLEND^®^ 4655V during recycling. The results of this work clearly show that it is very important to investigate changes in the properties of cellulose–acetate-based blends during multiple recycling cycles, especially across different processing temperatures, as the behaviour of the recycled material can alter even upon small temperature variations. A limitation of the present study is that no microstructural or thermal-degradation analyses (such as SEM of fractured surfaces or thermogravimetric analysis, TGA) were conducted. Consequently, the proposed degradation and branching mechanisms are inferred from rheological, DSC, and mechanical data rather than directly corroborated by morphological or mass-loss measurements. Future work should therefore include SEM and TGA in order to visualise damage features at the microscale and quantify the onset and kinetics of thermal degradation under repeated processing.

## Figures and Tables

**Table 1:** Injection moulding parameters.

Process Parameter	Value
Injection speed [s]	2.6
Cooling time [s]	15
Screw speed [mm/s]	200
Holding pressure [bar]	600
Holding time [s]	13
Back pressure [bar]	70
Mould temperature [°C]	30
Nozzle temperature [°C]	170/180/200
Temperature (Zone 3) [°C]	170/190/210
Temperature (Zone 2) [°C]	160/180/200
Temperature (Zone 1) [°C]	150/170/190
Temperature (Feed) [°C]	40

**Table 2:** Melting peaks, onset, offset, and enthalpy determined from the DSC second heating curve for virgin and recycled ARBOBLEND^®^ 4655V processed at different temperatures: of 170 °C, 190 °C and 210 °C.

Processing Temperature	Recycling Run	1st Peak [°C]	2nd Peak [°C]	Onset [°C]	Offset [°C]	Enthalpy [J/g]
		Mean (SD)	Mean (SD)	Mean (SD)	Mean (SD)	Mean (SD)
170 °C	R0	78.99 (0.82)	86.45 (0.33)	74.12 (1.45)	91.13 (0.58)	34.69 (0.03)
	R1	79.31 (1.19)	86.28 (0.20)	71.54 (0.37)	90.77 (0.95)	34.99 (0.11)
	R2	80.87 (1.88)	86.28 (0.15)	69.61 (0.90)	91.28 (0.57)	35.14 (0.02)
	R3	78.32 (0.18)	86.11 (0.01)	70.67 (0.01)	90.15 (0.21)	35.95 (0.02)
	R4	78.64 (0.16)	86.42 (0.33)	70.48 (0.21)	91.04 (0.27)	34.90 (0.73)
190 °C	R0	80.75 (1.27)	86.46 (0.34)	69.71 (0.20)	91.22 (0.33)	35.03 (0.24)
	R1	82.27 (1.13)	86.13 (0.34)	71.67 (0.38)	91.01 (0.41)	34.98 (0.28)
	R2	83.96 (0.85)	*	71.46 (0.09)	90.41 (0.17)	34.37 (1.11)
	R3	83.62 (1.8)	*	72.22 (0.01)	90.89 (0.56)	34.39 (0.12)
	R4	85.14 (0.02)	*	71.28 (0.76)	91.54 (0.16)	35.28 (0.80)
210 °C	R0	82.46 (0.44)	86.78 (0.65)	69.68 (0.28)	92.24 (0.22)	36.32 (1.07)
	R1	79.33 (0.50)	86.30 (0.17)	73.89 (1.40)	90.84 (0.47)	34.83 (0.14)
	R2	81.59 (1.07)	87.15 (0.33)	70.58 (0.04)	92.40 (0.37)	35.11 (0.36)
	R3	78.50 (0.34)	86.45 (0.34)	71.03 (0.10)	90.90 (0.41)	33.59 (1.80)
	R4	77.84 (0.66)	86.78 (0.34)	70.63 (0.17)	91.15 (0.79)	34.86 (0.06)

* monomodal.

**Table 3:** Crystallisation peaks, onset, offset, and enthalpy determined from the DSC second heating curve for virgin and recycled ARBOBLEND^®^ 4655V processed at different temperatures of 170 °C, 190 °C and 210 °C.

Processing Temperature	Recycling Run	Peak [°C]	Onset [°C]	Offset [°C]	Enthalpy [J/g]
		Mean (SD)	Mean (SD)	Mean (SD)	Mean (SD)
170 °C	R0	50.42 (0.51)	56.91 (0.20)	43.33 (0.32)	29.69 (0.03)
	R1	51.86 (0.44)	56.66 (0.23)	46.02 (0.88)	28.63 (0.21)
	R2	51.72 (0.31)	56.66 (0.06)	45.54 (0.47)	28.17 (0.19)
	R3	51.81 (0.17)	56.61 (0.03)	46.26 (0.16)	28.26 (0.11)
	R4	51.31 (0.00)	56.21 (0.05)	45.41 (0.11)	26.81 (0.67)
190 °C	R0	51.42 (0.83)	56.22 (0.01)	44.40 (0.72)	29.80 (0.07)
	R1	53.60 (0.34)	58.47 (0.17)	46.83 (0.50)	30.15 (0.43)
	R2	55.25 (0.00)	60.39 (0.04)	48.15 (0.25)	29.72 (0.21)
	R3	55.42 (0.16)	60.85 (0.23)	48.17 (0.52)	30.13 (0.02)
	R4	55.19 (0.02)	61.66 (0.13)	46.19 (0.31)	30.40 (0.10)
210 °C	R0	51.10 (0.16)	56.58 (0.09)	43.86 (0.15)	30.05 (0.26)
	R1	52.45 (0.17)	57.43 (0.09)	46.51 (0.50)	29.98 (0.04)
	R2	52.26 (0.33)	57.29 (0.13)	45.59 (0.39)	30.01 (0.15)
	R3	52.80 (0.17)	57.73 (0.12)	47.16 (0.43)	31.23 (0.63)
	R4	52.47 (0.18)	57.66 (0.00)	47.43 (0.00)	31.51 (0.55)

**Table 4:** Impact strength for the recycling cycles R0-R4 processed at 170 °C, 190 °C, and 210 °C.

Processing Temperature	Recycling Run	Number of Specimens	Impact Strength
**170 °C**	R0	5	N
	R1	7	N
	R2	5	N
	R3	7	N (59.38 ± 9.72 KJ/mm^2^ C two Samples)
	R4	4	N (47.13 ± 0.53 KJ/mm^2^ C two Samples)
**190 °C**	R0	5	N
	R1	5	N
	R2	5	N
	R3	5	N
	R4	5	N
**210 °C**	R0	5	N
	R1	5	N
	R2	5	72.75 ± 5.06 KJ/mm^2^ C
	R3	5	63.05 ± 5.39 KJ/mm^2^ C
	R4	5	46.1 ± 10.82 KJ/mm^2^ C

N = no break, C = complete break.

## Data Availability

The original contributions presented in this study are included in the article. Further inquiries can be directed to the corresponding author.

## Abbreviations

The following abbreviations are used in this manuscript:

ATR	attenuated total reflection
CA	cellulose acetate
DS	degree of acetyl substitution
DSC	differential scanning calorimetry
IR	infrared
M.W.	molecular weight
MFR	melt flow rate
MWD	molecular weight distribution
PA	polyamide
PCL	polycaprolactone
PE-HD	high-density polyethylene
PE-LD	low-density polyethylene
PET	polyethylene terephthalate
PHA	polyhydroxyalkanoates
PHBV	poly(3-hydroxybutyrate-co-3-hydroxyvalerate)
PLA	polylactic acid
PP	polypropylene
SEM	scanning electron microscopy
TGA	thermogravimetric analysis
